# Nutrition Care Practices of Dietitians and Oral Health Professionals for Oral Health Conditions: A Scoping Review

**DOI:** 10.3390/nu13103588

**Published:** 2021-10-13

**Authors:** Jessica R. L. Lieffers, Amanda Gonçalves Troyack Vanzan, Janine Rover de Mello, Allison Cammer

**Affiliations:** College of Pharmacy and Nutrition, University of Saskatchewan, Saskatoon, SK S7N 5E5, Canada; agv665@usask.ca (A.G.T.V.); jar018@usask.ca (J.R.d.M.); allison.cammer@usask.ca (A.C.)

**Keywords:** oral health, dental caries, diet therapy, dentists, dental auxiliaries, nutritionists, dietitian, surveys and questionnaires, qualitative research, review

## Abstract

Background: Oral health conditions, such as dental caries, pose a substantial burden worldwide. Although there are many risk factors for poor oral health, diet is often implicated as a cause of these issues. The purpose of this scoping review was to identify and map studies that have captured information on the “real-world” nutrition care practices of oral health professionals (OHPs) and dietitians to optimize oral health, and specifically the dentition and periodontium. Methods: A search of peer-reviewed articles was conducted using MEDLINE, CINAHL, and Embase. Articles that addressed the review objective and met the following criteria were included: English language, published since 2000, and study conducted in a high-income country. Results: Overall, 70 articles were included. Most articles reported on cross-sectional survey studies and provided self-reported data on OHP practices; few articles reported on dietitians. Most articles reported only general/unspecific information on assessment and intervention practices, such as dietary analysis, nutrition counselling, and diet advice, and lacked specific information about the care provided, such as the dietary assessment tools used, type of information provided, and time spent on these activities. Barriers to the provision of nutrition care by OHPs were common and included time and lack of remuneration. Few studies reported on collaboration between dietitians and OHPs. Conclusions: Several studies have captured self-reported information on nutrition care practices of OHPs related to oral health; however, there is limited information available on the details of the care provided. Few studies have examined the practices of dietitians.

## 1. Introduction

Worldwide, oral health issues (e.g., dental caries, periodontal disease) affect 3.5 billion people [1], with dental caries being the most common concern [2]. Oral health issues also affect many people in Canada. For example, the 2007–2009 Canadian Health Measures Survey (CHMS) reported that 57%, 59%, and 96% of 6–11-year-olds, 12–19-year-olds, and adults, respectively, had experienced dental caries [3]. Dental caries are also the most common reason for day surgery in children 12–59 months of age in Canada [4]. Periodontal issues are also common in Canada; according to the 2007–2009 CHMS, 21% of adults with teeth were found to have or previously had moderate or severe periodontal issues [3]. Various factors can affect the risk of oral health issues, including fluoride, oral hygiene (e.g., brushing, flossing), tobacco, and diet [5].

More specific to diet, both the physical and chemical properties of the foods we eat, as well as how the foods we eat are consumed (e.g., frequency, delivery) can have protective or detrimental effects on oral health (and specifically the dentition and periodontium) [6]. Fermentable carbohydrates (e.g., sucrose) have a known relationship with dental caries [7]; however, the relationship between oral health and diet is much more extensive [8]. For example, hard cheese; sugar alcohols (e.g., xylitol); diets rich in vegetables, fruits, whole grains, and high-quality proteins; and adequate spacing between eating occasions are thought to be protective against dental caries [6,8,9]. Acidic foods and beverages have also been previously linked with tooth erosion [10,11]. The relationship between diet and oral health has been summarized in several articles [6,8,9,10,12,13,14,15,16,17], and some organizations have released statements and guidelines on this topic [8,18,19,20]. We also know that many people in Canada have diet behaviours associated with poor oral health (e.g., high intakes of sugar-sweetened beverages [21]). In addition to diet being linked to various oral health issues, poor oral health can also have nutritional implications (e.g., children with early childhood caries can experience difficulties eating [22]). Importantly, the relationship between nutrition and oral health is important throughout the entire human lifespan [8,12].

The provision of nutrition care to optimize oral health is within the scope of practice of different health professions including dietitians and oral health professionals. In addition, healthy eating has been identified as a priority area by the World Health Organization when initiating and strengthening oral health programs [23,24]. To date, information on the current “real-world” practices regarding nutrition care for oral health (and specifically the dentition and periodontium) provided by dietitians and oral health professionals has been captured in different types of studies with different focuses; however, reviews on this topic are limited. A previous review article published in 2014 examined the diet advice practices of oral health professionals, including variables that influence this activity [25]. Since this article has been published, there have been several other relevant studies that have emerged. This article also did not examine the practices of dietitians. 

The purpose of this scoping review is to identify and map current studies that have captured information on the “real-world” nutrition care practices of oral health professionals and dietitians to optimize oral health (specifically the dentition and periodontium), in addition to collaboration between the two professions. We wanted to capture information on the scope of the literature addressing this topic and consolidate the wide range of studies to identify gaps in the literature to understand how to move this area of research forward. This project lends itself well to a scoping review because of the diversity of studies and study designs that have investigated this topic. This review did not seek to investigate the impact of different types of nutrition care practices on oral health.

## 2. Materials and Methods

The scoping review framework outlined by Arksey and O’Malley [26] was used to guide this review. In addition, the article by Levac et al. [27] was also used to help guide this review. The five steps that were followed to conduct this review are outlined below. A review protocol does not exist for this scoping review.

Stage 1: Research Question Development

The first component of conducting a scoping review is to identify the research question that will be investigated. The question that was used to guide the review was as follows: What is reported in the literature about the “real-world” practices of dietitians and oral health professionals regarding nutrition care for oral health (and specifically conditions that affect the dentition and periodontium)? In addition, we were also interested in understanding what has been studied regarding collaboration between the two professions for this purpose. According to the FDI World Dental Federation definition, “oral health is multi-faceted and includes the ability to speak, smile, smell, taste, touch, chew, swallow and convey a range of emotions through facial expressions with confidence and without pain, discomfort and disease of the craniofacial complex (head, face, and oral cavity)” [28]. We were specifically interested in studies focusing on the health of the dentition and periodontium. A scoping review for this research question is suitable as we were interested in understanding the range of the evidence to date, and because there are numerous possible study designs that could address the research question. The purpose of this review is to help to summarize the extent and nature of the research activity in this area. This review will help to identify and clarify research needs to help advance the area of nutrition care and oral health (specifically conditions that affect the dentition and periodontium) to help decrease the burden of oral diseases in Canada and beyond.

Stage 2: Identification of Relevant Studies

A literature search was conducted using the MEDLINE, CINAHL, and Embase databases in May 2020 and repeated in May 2021 (as there were delays completing this review due to the COVID-19 pandemic). Health science academic librarians provided guidance on the database searches. Three categories of concepts that were derived from the research question were used in the literature search. The first category related to oral and dental health, dentistry, and oral health professionals. The second category related to dietitians, nutrition services, dietetics, food, nutrition, and diet. The third category related to professional practices and behaviours. The MEDLINE search strategy is provided in Table 1.

In addition to database searches, reference lists from all included articles were examined for additional articles. Moreover, the citations for relevant articles were searched in Google Scholar for any other papers that might have been missed. Relevant review articles in this area were also consulted to capture any additional articles [25,29,30]. Hand searches of journals likely to have articles in this area (Journal of Dental Hygiene, International Journal of Dental Hygiene, Community Dental Health, Canadian Journal of Dental Hygiene) were also conducted. All articles were imported into a reference management system and duplicates were removed. 

Stage 3: Article Selection

Article inclusion and exclusion criteria are listed in Table 2. In order to be included, articles had to meet the following criteria: English language, published in year 2000 or later, conducted in a World Bank high-income country, reported information on the “real-world” practices of dietitians/nutritionists and/or oral health professionals (not including students) regarding nutrition care for oral health (and specifically conditions affecting the dentition and periodontium). Articles were included regardless of quality. Clinical trials were not included except for baseline information on current professional practices. The year 2000 was chosen to select articles that were relatively recent. World Bank high-income countries were chosen to provide articles that are most relevant to the Canadian context. Exclusion criteria included information on the practices of students (e.g., nutrition students, dental students, dental hygiene students), where results of dietitians/nutritionists and/or oral health professionals were combined with other professionals (e.g., nurses, physicians) and could not be isolated, information on professional commentary regarding treatment plans for case studies, and when information about nutrition care practices was combined with other dental prevention practices (and cannot be isolated) (e.g., dental preventative activities in general were assessed which could include nutrition interventions amongst other activities such as fluoride application). Studies that were conducted in Special Supplemental Nutrition Program for Women, Infant, and Children (WIC) personnel were included as well. WIC is a federally funded government program in the United States that is available for low-income pregnant women, postpartum breastfeeding and nonbreastfeeding women, and children less than 5 years of age [31]. This program provides healthy foods, nutrition education, and health care referrals. Of note, many WIC staff members are also dietitians. We also included studies that included information on practices related to oral health (e.g., tooth brushing instruction) in dietitians, nutritionists and/or WIC personnel. 

Two individuals independently screened the titles and abstracts of all articles from the search conducted in May 2020 using the study inclusion and exclusion criteria with Rayyan [32]; if the abstracts did not provide enough information, the full text of the article was consulted. For the articles from the search conducted in May 2021, the articles were also screened by two individuals using the study inclusion and exclusion criteria. Cases of disagreement were reviewed and discussed by a third member of the research team until consensus was reached.

Stage 4: Charting the Data

The following information was charted from all eligible articles: author(s), year of publication, study location, study dates, study population, sample size, study methodology, oral health issue of focus, and key findings. Only information relevant to the inclusion criteria was extracted; for example, if a study included information about physicians and oral health professionals, only the information about oral health professionals was charted. The source of funding for the included studies was also captured. As with many scoping reviews, a quality assessment was not carried out. 

Stage 5: Collating, Summarizing, and Reporting the Results

Following extraction of all data, the results were collapsed into tables to report information on sample size, type of professional studied, patient population and/or concern of focus, and information about the topics investigated in the article. This process was conducted by JRLL, and the content was verified by AGTV or JRdM by looking at the content of the table and cross-checking it with the article. The articles were sorted based on the type of professional studied, the types of outcomes reported, and the study methodology. 

## 3. Results

**Figure 1:**
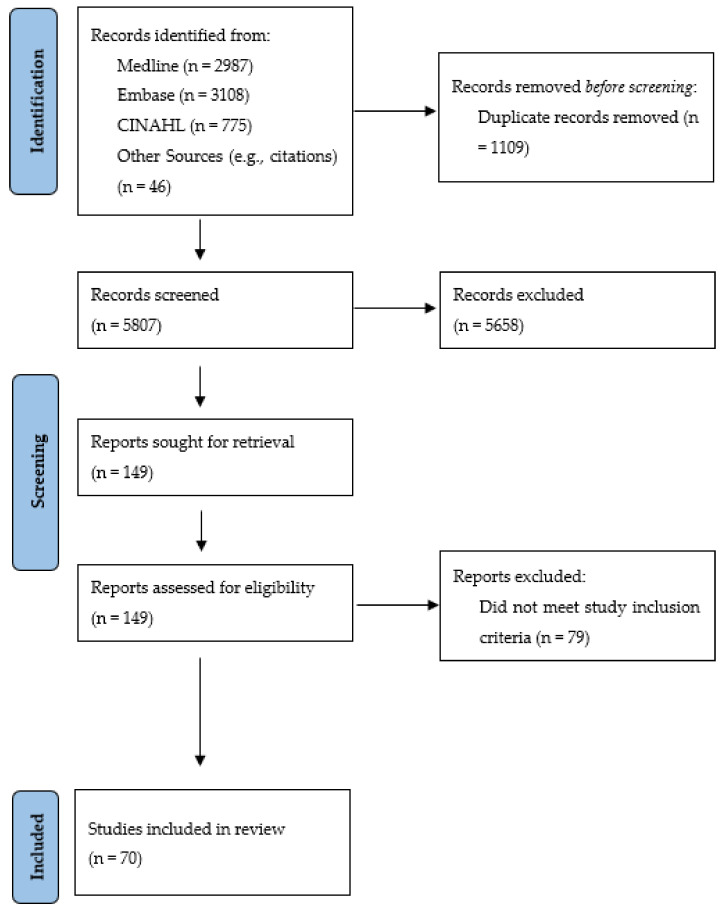
Search results.

In total, *n* = 70 articles were included in the scoping review (Figure 1). Most articles describe studies that were conducted in one of the following four regions: United States (*n* = 23), United Kingdom (*n* = 17), Nordic countries (Norway, Finland, Iceland, Sweden, Denmark) (*n* = 12), and Australia (*n* = 7). However, there were also articles from Japan (*n* = 3), Germany (*n* = 2), Saudi Arabia (*n* = 2), Canada (*n* = 1), Hong Kong (*n* = 1), New Zealand (*n* = 1), and Taiwan (*n* = 1). In total, *n* = 32 articles were published in 2010 or earlier and *n* = 38 articles were published in 2011 or later. Almost half of the articles did not report any funding source (*n* = 32); articles reporting funding sources (*n* = 38 articles) reported that their studies were funded by various sources including government/government agency (*n* = 25 articles), university/hospital (*n* = 6), professional association/organization (*n* = 3), and other sources (i.e., foundation, charity, company) (*n* = 10).

Most studies focused on oral health professionals; only six studies provided information on dietitians, nutritionists, and/or WIC personnel (many of whom are dietitians or nutritionists). Most articles did not focus specifically on diet and nutrition but rather had focuses including preventative dentistry, prevention and/or treatment of specific oral health conditions, infant oral health care/examinations, dental practice patterns, and public dental service use. Only *n* = 18 articles referred to something that was obviously related to nutrition and/or diet in their title (e.g., diet, nutrition, sugar-sweetened beverage, dietitian, nutritionist, WIC). Most included articles were cross-sectional survey studies of professionals (sample size range: *n* = 9 to *n* = 2294 professionals); however, other study methods included chart reviews (sample size range: *n* = 285 to >10,000 patients), observation studies (sample size: *n* = 3751 patient visits in 120 general dental practices), and qualitative interviews and/or focus groups of professionals (sample size range: *n* = 10 to 93). In addition, one study captured information on how different types of data compared to one another.

In this review, the articles were organized based on the type of professional studied, the methods used in the study (e.g., survey, qualitative interview), and types of care provided which encompassed either assessment practices (i.e., dietary assessment practices by oral health professionals; assessment practices regarding oral health and nutrition by dietitians, nutritionists, and/or WIC personnel) or intervention practices (e.g., diet counselling, diet advice by oral health professionals; intervention practices regarding oral health and nutrition by dietitians, nutritionists, and/or WIC personnel). Of note, two cross-sectional survey studies included only a combined measure of assessment and intervention practices; these articles are included in both of those sections.

### 3.1. Assessment Practices

#### 3.1.1. Oral Health Professionals

Nineteen articles provided information on the dietary assessment practices of oral health professionals. These studies are described in Table 3. Overall, most of these articles were cross-sectional survey studies usually of dentists; however, other types of studies also captured this information, including chart reviews and a qualitative interview study. Overall, these studies captured information on various dietary assessment practices including general/unspecific assessment practices (e.g., dietary analysis), use of specific diet assessment tools (e.g., food records), and inquiries about specific patient behaviours or concerns (e.g., bottle use, cariogenic food consumption). In addition, two studies examined barriers regarding dietary assessment. A few of these studies examined whether there were differences in these outcomes by professional type, patient type, and various professional and practice factors. 

##### General/Unspecific Dietary Assessment Practices

Thirteen articles reported information on general/unspecific dietary assessment practices (e.g., dietary analysis, dietary history, assessment of dietary habits, asking about dietary behaviour, diet history review and analysis) of oral health professionals (cross-sectional survey studies (*n* = 10) [33,36,38,39,41,42,44,46,47,48]; chart review studies (*n* = 2) [52,53], qualitative interview study (*n* = 1) [54]). Most of the articles reported information regarding these practices in dentists.

Within the cross-sectional survey studies, various self-reported measures were used to capture information on general/unspecific dietary assessment practices. One self-reported measure used in some of these studies was the proportion of respondents who provide this type of service in the population of interest [36,44]. These studies were conducted in pediatric dentists and found that this activity was common in the population of interest (e.g., >90% of pediatric dentists who performed infant oral health evaluations reported this practice [36]; 70.6% of pediatric dentists reported doing a diet history review and analysis regarding dental caries prevention [44]). Several cross-sectional survey studies also reported self-reported information on frequency of providing some type of general/unspecific dietary assessment practice [33,38,39,42,46,47,48]. Overall, these studies were conducted regarding these practices when assisting patients with specific dental conditions (e.g., root caries [39], erosive tooth wear [38,46,47,48]) and in children [33,42] and adult [42] patient groups. In articles that focused on specific dental conditions, the percentage of respondents who self-reported always, often, and/or usually providing some type of general/unspecific dietary assessment practice ranged from 45% to 81%, and the percentage of respondents who never or seldom performed this practice ranged from 1% to 25% [38,39,46,47,48]. In studies focusing on children and/or adults, the percentage of respondents who self-reported never performing this practice ranged from 2% to 6.4% [33,42]. The chart review studies that assessed general/unspecific dietary assessment practices found results that are slightly different compared to results found in the survey studies. These studies, which included one focusing on pediatric patients at high risk for developing dental caries in a public dental service [52] and another focusing on tooth wear in adults in general dental practice [53], found that <25% of patients had this type of assessment documented in their dental records.

The studies of Widström et al. [42] and Kangasmaa et al. [47] were the only studies that compared general/unspecific dietary assessment practices amongst different types of oral health professionals. Widström et al. [42] found in their survey study that dental hygienists were significantly more likely to assess dietary habits in all or most of their patients vs. dentists in both children and adult patients. Kangasmaa et al. [47] found regarding erosive tooth wear that there was no difference in the frequency of assessing a patient’s dietary history between general dentists and different types of dental specialists. In addition, four studies [33,41,47,53] examined professional and practice factors that impact the frequency with which dentists provide general/unspecific dietary assessment services for their patients. Overall, these studies found that dentists who were significantly more likely to practice this activity were less experienced (vs. more experienced) [53], work as a community dental officer (vs. general dental practitioner) [33], and have positive attitudes regarding prevention [41].

Three studies examined whether patient-related factors were associated with whether oral health professionals perform general/unspecific dietary assessment activities [42,52,54]. Widström et al. [42] found in their survey study that dentists and dental hygienists were more likely to perform an assessment of dietary habits in children compared to adults (e.g., % of dentists and dental hygienists who perform an assessment of dietary habits in all or most of their child patients: 18.9; % of dentists and dental hygienists who perform an assessment of dietary habits in all or most of their adult patients: 8.3). Threlfall et al. [54] found in their qualitative interview study of general dental practitioners that they were more likely to ask questions about a child’s diet if the child had dental caries.

Garton and Ford [39] investigated barriers reported by dentists regarding performing dietary analysis for root caries. They found that 43.3% of respondents reported barriers to providing dietary analysis. Time was the most common barrier (33.9% of respondents); however, other barriers encountered by <10% of respondents included cost, lack of usefulness, lack of authority to order, and dietary analysis not being evidence-based. In addition, these authors found statistically significantly more dentists working in the public sector vs. the private sector reported the barrier of lack of authority to order (public: 8.7% vs. private: 1.7%).

##### Use of Specific Dietary Assessment Tools

Six studies provide information on self-reported use of specific dietary assessment tools (e.g., diet diary) by oral health professionals (cross-sectional survey studies (*n* = 5) [35,38,43,46,48]; qualitative interview study (*n* = 1) [54]); of note, all of these studies were conducted in dentists. Overall, the survey studies found that less than 50% of dentists self-reported using some type of diet diary with their patients (e.g., 9% of pediatric dentists [35]; 28% of general dental practitioners [43]; 12–31% of dentists when referring to care of patients with erosive tooth wear [38,46,48]). These studies also found that less structured diet assessment approaches (e.g., oral interviews, asking patients to recall usual activities for a specified period) tend to be more commonly used by dentists compared to diet diaries [38,43,46,48]. 

Arheiam et al. [43] studied the use of diet diaries in UK general dental practitioners, including reasons for using diet diaries, factors that are considered when deciding to use these tools, routines when using diet diaries, and reasons for not using diet diaries. Of note, they found that diet diaries were more commonly used in children vs. adults, and the top reasons for lack of use of diet diaries were inadequate remuneration by the National Health Service (NHS), lack of usefulness, lack of knowledge, and poor compliance. The authors also found statistically significant predictors of diet diary use in a multivariate model were years of service, percentage of NHS patients in practice, and percentage of case-mix children in practice. The authors also reported the median time required to complete a diet diary analysis was 10 min (range: 1–23 min) and that patients were asked to keep a diary for a median of 3 days (range: 1–7 days) [43]. Lastly, a qualitative interview study by Threlfall et al. [54] found that a few general dental practitioners had attempted to use diet sheets in children, but reported little success. 

##### Inquiries about Specific Patient Behaviours or Concerns Related to Diet

In total, four cross-sectional survey studies report information on inquiries by oral health professionals about specific patient behaviours or concerns related to diet [34,37,40,45]; three of these studies were conducted in dentists [34,40,45] and one was conducted in dental hygienists [37]. Three of these studies captured self-reported information on asking about specific behaviours related to dental caries in children (e.g., between-meal exposure to food that causes dental caries, bottle use, juice consumption) [37,40,45], and one study focused on older adults [34].

Overall, the studies that captured information on inquires about specific behaviours in children related to diet and dental caries found that this practice is relatively common [37,40,45]. For example, Clovis et al. [37] found that among dental hygienists who routinely assess their child/youth patients for risk factors related to dental caries (88.7% of respondents), 64.9% self-reported asking about between-meal exposure to cavity-producing foods. In addition, Sim et al. [40] found that the frequency of asking about different feeding practices (including bottle feeding and juice consumption) in infants and toddlers can vary depending on the practice (e.g., 77% of respondents reported asking about bottle contents all of the time vs. 27% of respondents reported asking about age of first juice consumption all of the time). One study captured differences in these types of practices between different types of oral health professionals. Dima et al. [45] found that a significantly higher percentage of pediatric dentists self-report asking about bottle use often or very often compared to general dentists (94.9% vs. 67.2%).

In general dentists, Hawkins and Locker [34] found that asking about different types of behaviours relevant to eating in older adults varied depending on the behaviour (e.g., 55% of respondents usually asked about prevention from eating desired foods because of chewing problems vs. 15% of respondents usually asked about avoiding eating with other people because of chewing problems). These authors [34] also found that numerous practice and dentist demographic characteristics were significantly related to inquiring about various behaviours relevant to eating in older adults in bivariate (and for some variables multivariate) analyses including dentist involvement in taking patient history, self-rated ability to treat older adults who reside in institutional settings, estimated percentage of patients 65+ years of age, continuing education in geriatric dentistry in the last year, time taken to obtain a patient history, dentist age, population size of primary practice location, and dental school experience in geriatric outreach care.

#### 3.1.2. Dietitians and Nutritionists

Three cross-sectional survey studies captured self-reported information about assessment practices of dietitians and/or nutritionists (including WIC personnel) related to nutrition and oral health or oral health only [49,50,51]; these studies are also described in Table 3. Fuller et al. [49] found that less than 50% of surveyed WIC personnel assessed children for visual evidence of dental caries; they further found that respondents ≥40 years of age were significantly more likely to do this practice compared to respondents 18–39 years of age (53% vs. 28%). Gold and Tomar [50] found in a small study of WIC nutritionists that 33% of respondents frequently asked about women’s and caregivers’ dental health and no respondents reported frequently examining the teeth of children for dental caries. They also found that 100% of respondents frequently asked about whether the child brought a bottle to bed. In addition, Fernandez et al. [51] found in dietitians who had completed an elective pediatric dentistry rotation during their dietetic internship training that 28% always and 31% never collect information on an adult’s oral health history and 24% always and 38% never collect information on a child’s oral health history.

### 3.2. Intervention Practices

#### 3.2.1. Oral Health Professionals

Nutrition intervention practices (e.g., diet counselling, nutrition counselling, dietary advice, dietary instruction, providing information on diet, nutrition advice) in oral health professionals were investigated in *n* = 48 studies (presented in *n* = 53 articles); these studies are described in Table 4. Overall, most articles reported on cross-sectional survey studies (*n* = 36 studies presented in *n* = 39 articles). In addition, there were *n* = 8 chart review studies (one of which had professionals keep detailed documents of encounters over a 2-week period), *n* = 1 observation study that also included a survey and chart review study (presented in *n* = 2 articles), and *n* = 3 qualitative interview/focus group studies (presented in *n* = 4 articles) that were included. In general, most of the studies provided information on general/unspecified nutrition intervention practices (e.g., nutrition counselling, diet advice); however, there were also studies that discussed types of resources/strategies used, information provided to patients, and barriers regarding intervention practices. 

##### General/Unspecific Nutrition Intervention Practices

In total, *n* = 33 cross-sectional survey studies presented in *n* = 36 articles, *n* = 8 chart review studies, *n* = 1 observation study presented in *n* = 2 articles, and *n* = 1 qualitative interview study presented in *n* = 2 articles provide information on the delivery of general/unspecific nutrition interventions (e.g., diet counselling, nutrition counselling, dietary advice, dietary instruction, providing information on diet, nutrition advice) by oral health professionals. In general, most of the survey studies that captured self-report information on this topic report on the proportion of oral health professionals providing this service with or without some type of measure of how often they provide this service. Of note, most of these studies reported information on dentists; however, there were eight studies that also captured information on the practices of other oral health professionals (e.g., dental hygienists, dental therapists, dental nurses) [59,64,66,67,69,78,82,84]. Overall, most of these studies find using a variety of different types of measures that many oral health professionals self-report carrying out nutrition interventions at least some of the time. However, within these survey studies, only a few studies captured information on the percentage of patients receiving this care and found it varied from a mean of 21.4% to 63.0% [43,74,75,76]. In addition, a few survey studies reported that the length of time spent on these activities was brief (approximately five minutes) [35,60]. 

In contrast to the cross-sectional survey studies which provide self-report data, the chart review studies (one of which includes professionals documenting all preventative activities over a 2-week time frame) and observation studies generally find that this practice is variable and often limited. For example, these studies conducted in children and adolescents have found that nutrition interventions are provided to a range of <10% of children/adolescents to >50% of children/adolescents in both public dental service and other practice settings [91,92,93,94,97], that dietary advice represented <10% of clinical activities in public dental service [95], and that the number of diet instruction sessions is low in public dental clinics (e.g., ≤~1 per child/adolescent in the observation periods) [90]. Of note, the study by Sarmadi et al. [94] also found that diet counselling was provided to ~10–20% of children and adolescents whereas diet information was provided to ~40–50% of children and adolescents attending public dental services in Sweden. In addition, Raindi et al. [96] found in a small pilot study that diet advice was not provided for periodontal prevention. As well, an observation study [98,99] conducted in general dental practices in the United States found that overall <~10% of visits had nutrition counselling with the median number of 30 s intervals devoted to nutrition counselling being 0 (range: 0–6 for dentists; 0–23 for dental hygienists). In addition, this study also found that among dentists and dental hygienists who self-reported often/always providing a nutrition discussion using a self-administered survey, only a mean of 3.9%, and 13.7% of the patients of these dentists and dental hygienists, respectively, were observed to actually receive this service. In addition, when this practice occurred, <5% of visit time was spent on this activity. 

Very few studies provide information on how general/unspecific nutrition intervention practices of different types of oral health professionals compare to one another [63,71,82,85,87,98,99]. Overall, most of these studies found that pediatric dentists more commonly provided these interventions to infant patients compared to other dentists [63,71,85,87] and that dental hygienists and/or oral health therapists provide these interventions more frequently compared to dentists [82,98,99]. 

In total, 11 studies [54,57,59,78,90,91,92,93,94,95,97] examined the provision of general/unspecific nutrition interventions by patient type. Six studies [57,59,90,91,92,93] found that the provision of general/unspecific nutrition interventions generally increased as the severity of dental disease (e.g., dental caries, erosion), risk of dental disease, or amount of dental care increased; however, only three studies reported significant results [90,91,93]. Studies also found that in children and adolescent patients, those who are younger (vs. older) [95], those who are less disadvantaged when looking at socioeconomic status (vs. more disadvantaged) [97], and patients/families who the professional felt were more motivated and with whom they had seen success [54] were more likely to receive nutrition interventions from oral health professionals. In addition, one study found that the frequency of delivery of dietary advice by dental nurses was similar for children <2 years of age compared to children >2 years of age [78].

Seventeen survey studies presented in 19 articles investigated various professional and practice factors that impact the provision or the intent to provide general/unspecific nutrition interventions, with some of these studies finding statistically significant results. First, some studies found that oral health professional demographic variables showed some type of statistically significant relationship with the provision of general/unspecific nutrition interventions, including oral health professional age [79]; sex [73,75,79,86]; knowledge, skills, and/or education in this area [78,84]; and ownership of practice [73]. Studies have also found that attitudes, perceived behavioural control, motivation, and/or confidence in this area were statistically significantly associated with providing nutrition interventions [40,41,60,75,78,83]. A study of dental hygienists found that working more hours per week was associated with increased likelihood of providing nutrition counselling [69]. Studies also found that practice characteristics were statistically significantly associated with providing general/unspecific nutrition interventions, including prevention focus/orientation [75,76], practice location (e.g., rural vs. urban) [66], practice constraints [40], practice busyness [75], and practice type and setting (e.g., private vs. public; community dental service vs. general dental practice) [33,65,67,86]. Moreover, a chart review study of public dental services found that rural health districts provided less dietary advice compared to metropolitan health districts [95]. Lastly, Threlfall et al. [54] found in a qualitative study of general dental practitioners regarding care of children with dental caries that practices with a dental hygienist had an increased focus on providing diet advice.

##### Types of Resources/Strategies Used

Only a few studies [43,54,55,60,83,84] reported on types of specific nutrition education materials, resources, and/or strategies used when oral health professionals provide nutrition interventions. Most of the studies that have investigated this topic have been cross-sectional survey studies that provide self-reported information. Chisick et al. [55] and Huang et al. [60] found that oral presentations/oral discussions and paper fliers/handouts were the most common ways to deliver this information. Huang et al. [60] reported on the frequency of providing nutrition advice to parents compared to children, and they found similar results between the two groups. In addition, Wright and Casamassimo [83] investigated in pediatric dentists and pediatric dental residents the frequency of using different types of intervention strategies to reduce consumption of sugar-sweetened beverages; they found that speaking to parents about observations if the child has high risk of dental caries was the most common strategy, followed by documenting the high risk of dental caries in the patient’s chart. Provision of educational materials on sugar-sweetened beverages and offering motivational interviewing or other behaviour modification programs were also relatively common strategies. They further found that less common intervention strategies included providing parents with a self-administered sugar-sweetened beverage screening tool, and following up on interventions. Cole et al. [84] examined the frequency of provision of different types of nutrition interventions by dental hygienists. They found that nutrition counselling was more common than advocacy and collaborative activities (e.g., advocating to school officials to ensure healthy foods are available in school food services). For advocacy and collaborative activities, they also found that those with continuing education on obesity were more likely to do those activities compared to those who had obesity education only within an entry-level dental hygiene program. Arheiam et al. [43] also found that 40% of general dental practitioner respondents referred patients to other oral health professionals (e.g., dental hygienists) for advice on diet for an average of 11% of their patients. Lastly, Threlfall et al. [54] found in a qualitative study of general dental practitioners regarding caring for children with dental caries that oral advice was the most common way to provide dietary information, but providing leaflets was also done. They also found that general dental practices that had a dental hygienist often had preventative activities (e.g., diet advice) delegated to those professionals. 

##### Types of Nutrition Information Provided

Few details on the types of nutrition information provided to patients by oral health professionals were present in the included articles; only 10 survey studies reported this type of finding [35,55,56,57,60,63,64,83,84,87]. Topics that were captured in the survey studies included nursing caries/baby bottle decay risk [55,63,87]; consumption of sugary foods and drinks (including timing) [35,56,83,84]; consumption of between-meal snacks [84], supplements (including recommending vitamins) [35,64]; and consumption of erosion-causing foods, specifically carbonated soft drinks, fruit juice, acidic drinks, and citrus fruit [57]. In addition, Huang et al. [60] provided information on the proportion of orthodontists who provided patients handouts on foods to avoid (56% of respondents) and foods to encourage (32% of respondents). 

Very few studies examined differences between different types of oral health professionals for this type of finding. Brickhouse et al. [63] found that discussing baby bottle decay risk during infant oral health exams was common and similar between pediatric dentists and general dentists (pediatric dentists: 98%; general dentists: 100%). In addition, Bakhurji et al. [87] found that 60% of pediatric dentists and 64% of general dentists provided nutrition counselling and talked about baby bottle decay in infant oral health care. The work by Dugmore and Rock [57] was the only study to examine the proportion of dentists who provided specific dietary messages in different patient populations. They reported the proportions of dentists who provided advice on carbonated soft drinks, fruit juice, acidic drinks, and citrus fruit for patients presenting with tooth erosion into (a) the enamel and (b) the dentine. The percentage of dentists who gave the specific types of advice ranged from 6.6% to 31.7% depending on the food/beverage topic and whether the tooth erosion was into the enamel or dentine.

A qualitative interview study by Threlfall et al. [54,100] provided in-depth information on the content of dietary advice provided by general dental practitioners to children who are dealing with dental caries and their parents. In this study, all general dental practitioners reported providing dietary advice. Dietary advice was primarily centred on decreasing consumption of sugar, and sugar intake frequency was often considered a key message. Sugary beverages were also a common topic for dietary advice. The authors reported that there were variations in dietary advice content and emphasis (e.g., some participants provided advice on extrinsic sugars while others did not; some participants said to limit carbonated drinks due to erosion, and others said to reduce intake of these drinks because of sugar). Some participants also reported that they emphasized oral hygiene instead of diet because they felt it was easier to change, and some participants also reported trying to provide advice that was practical knowing that children like sugary foods and beverages (e.g., providing strategies on how to consume sugar-rich foods to decrease risk of caries). The authors also found that participants tailored the advice to parents based on their perception of the level of ignorance.

##### Barriers Regarding Intervention Practices

In total, six survey studies [40,60,61,80,82,83] examined the prevalence of barriers associated with the provision of nutrition interventions by oral health professionals using quantitative methods; five studies examined these barriers in dentists [40,60,61,82,83], and three studies examined these barriers in other types of oral health professionals (i.e., dental hygienists, oral health therapists) [61,80,82]. The most common barriers studied were compensation, funding, and/or reimbursement [40,60,61,80,82,83]; time [40,60,61,80,82,83]; lack of professional training, knowledge, and/or skills [40,61,80,82,83]; and patient motivation, compliance, and/or interest (including difficulties in changing behaviour) [40,80,83]. Other barriers examined in two studies included patient knowledge and demographics (e.g., literacy, education level, socioeconomic status) [80,82]; lack of trained staff [40,83]; lack of resources [60,83]; communication, language, and/or cultural barriers [60,82]; lack of likelihood of effectiveness or benefit [61,82]; and fear of judging, alienating, and/or offending patients [61,83]. Of note, time was among the top three most commonly reported barriers in four of those studies [60,61,80,82]. Additionally, lack of patient motivation, compliance, and/or interest (including difficulties in changing behaviour) [40,80,83]; compensation, funding, and/or reimbursement [40,61,82]; and lack of professional training, knowledge, and/or skills [61,80,82] were among the top three most commonly reported barriers in three of those studies.

Dyer and Robinson [61] examined differences in the prevalence of perceived barriers reported by principal dentists to providing advice on diet/calorie intake by dentists and professionals complementary to dentistry (PCDs). The authors found statistically significant differences in the following barriers between the two types of professionals: time (dentists: 30.7%; PCDs: 16.9%) and lack of training/knowledge (dentists: 22.3%; PCDs: 7.8%). The authors found no differences for other investigated barriers (i.e., funding, effectiveness, patient alienation). 

Two studies examined the relationship between barriers and implementation of nutrition interventions in pediatric dentists. Sim et al. [40] found that when practitioners were grouped into those who provide dietary recommendations all of the time and those who do not, those who provide dietary recommendations all of the time had a significantly lower proportion of respondents who reported the following barriers: infant/toddler oral health is not a practice focus, recommendations are confusing/ambiguous, deficiency of trained auxiliaries, and time constraints. Wright and Casamassimo [83] found in pediatric dentists and dental residents that there was a significant relationship between provision of interventions regarding sugar-sweetened beverages and several different barriers, including reimbursement, time, lack of knowledge/skills, concern about offending patients, lack of educational materials, and legal concerns. 

In addition, three articles captured qualitative data using various methods (e.g., interviews, focus groups, open-ended survey questions) on barriers to providing nutrition interventions from the perspectives of oral health professionals [54,64,101]. Lack of oral health professional confidence and knowledge [64,101], lack of evidence-based guidance [64], funding and/or time [54,64], and lack of patient motivation and knowledge [54] were all identified.

#### 3.2.2. Dietitians and Nutritionists

Four cross-sectional survey studies examined provision of interventions for oral health provided by dietitians or nutritionists (including WIC personnel) [49,50,51,89]. Importantly, three of these studies were conducted in WIC personnel [49,50,89].

First, Butani et al. [89] assessed how often oral health was discussed by WIC personnel with clients. In total, they found that 37% of respondents discussed oral health issues most of the time or every time and 21% of respondents discussed it none of the time or a little of the time. They also found that 69% of respondents discussed oral health with more than 50% of their clients. Nursing training and oral health training were significant predictors for WIC personnel to discuss oral health with their clients. Fuller et al. [49] found that a significantly higher percentage of WIC personnel from rural districts self-reported advising parents/guardians on fluoride treatments or supplements compared to those from urban districts (rural: 58%; urban: 42%). Tooth brushing counselling provision in WIC clients by WIC personnel was assessed in two studies. Fuller et al. [49] found that more than 50% of surveyed WIC personnel provide tooth brushing counselling and that it was significantly related to practitioner years of experience. In addition, Gold and Tomar [50] found that 67% of WIC nutritionists frequently advise caregivers on the significance of frequent tooth brushing for their child. They also found that 67% of WIC nutritionists frequently advise caregivers on the importance of dental visits and 11% frequently discuss how the women’s oral health and the child’s oral health are linked. Gold and Tomar [50] also found that 100% of respondents frequently talk about how sugary drinks and snacks have a role in dental caries.

Only one study examined the intervention practices of dietitians outside of a WIC setting related to oral health. Fernandez et al. [51] surveyed dietitians who completed an elective pediatric dentistry rotation during their dietetic internship training. This study found that among participants, 20% always and 20% never include information about oral health as part of diet counselling; 40% always and 17% never consider the impact on oral health when recommending healthy foods; 34% always and 14% never consider the impact on teeth when providing counselling regarding sugary beverages. The authors also found that 17% always and 34% never provide education on oral health.

The study of Fernandez et al. [51] was the only study that provided information on the perceived barriers of dietitians in providing their services to dental patients. They investigated different types of barriers, and their results are as follows: cost/reimbursement of dietitian services (significant barrier: 57% of respondents; possible barrier: 43%), interest of clients in diet and oral health (significant barrier: 29%; possible barrier: 63%; not a barrier: 9%), dentist reluctance to refer their patients to a dietitian (significant barrier: 37%; possible barrier: 51%; not a barrier: 11%), and dietitian confidence in providing counselling and/or information in this area (possible barrier: 62%; not a barrier: 38%).

#### 3.2.3. Collaboration between Dietitians and Oral Health Professionals

Cross-sectional survey studies of oral health professionals investigated collaboration between nutrition/dietetics and dentistry using various outcomes, including referrals to dietitians/nutritionists [35,60,83,84], nutrition referrals [55,60], and/or recommendation of dietetics [81]. Although slightly different outcomes were used, they reported that the majority of oral health professionals never provide referrals to dietitians/nutritionists.

Four survey studies examined referrals to dentists by dietitians, nutritionists and/or WIC personnel [49,50,51,88]; three of those studies were conducted in WIC personnel [49,50,88]. For example, Gold and Tomar [50] found that 44% of WIC nutritionists frequently referred children or women to dental care. Fuller et al. [49] found that a significantly higher percentage of WIC personnel from urban districts referred clients to a dentist compared to those from rural districts (urban: 54%; rural: 46%). Shick et al. [88] comprehensively studied the dental care referral practices of WIC personnel. The authors found that 95.6% of respondents reported making dental referrals for children 1–5 years, and of those, 52.3% conducted this activity very frequently or frequently; referrals for infants were much less common. These authors also found that statistically significant predictors of referring children aged 1–5 years to dental care included older practitioner age (i.e., ≥40 years vs. younger), higher frequency of seeing dental concerns in patients (vs. lower), higher frequency of parents asking about obtaining dental care for their children (vs. lower), higher confidence in performing oral health risk assessment (vs. lower), higher confidence in making dental referrals (vs. lower), and higher confidence that patients will access dental care if advised (vs. lower). This study also reported that the most often used referral locations were local health departments (60.3% of respondents) and private dental offices (31.7% of respondents). The study of Fernandez et al. [51] was the only survey study that examined referrals to oral health professionals by dietitians outside of a WIC setting. The authors found that 54% of dietitians referred patients to pediatric or general dentists in their practice. 

The qualitative study by Ong et al. [102] examined interdisciplinary collaboration between dietitians, dentists, and physicians in Hong Kong in regards to diet. The authors found that barriers included those associated with electronic health records (e.g., not used by all the different professions), limited contact between the professions, lack of financial coverage, and inconsistent diet advice between different professions due to a lack of understanding of the advice given by others and different focuses of treatment administered by the different professions. Ideas presented to facilitate collaboration included more collaboration in undergraduate education, interprofessional education events, and development of guidelines with input from different professions. 

## 4. Discussion

To our knowledge, this is the first scoping review to comprehensively examine the literature on the nutrition care practices of oral health professionals and dietitians/nutritionists to optimize oral health (and specifically conditions that affect the dentition and periodontium). This review provides valuable information on the types of published evidence available in this area and a summary of key findings of these studies. The information gathered through conducting this review helps to provide direction on future strategies needed to move this area of research forward, which is important as oral health issues are prevalent in Canada and worldwide.

Although there appears to be substantial interest in this topic due to the volume of studies published that report information in this area, most articles provide only general and unspecific findings (e.g., capturing how often oral health professionals provide dietary advice) and focus mainly on oral health professionals (and especially dentists). Most of the data are also self-reported, which has limitations. Moreover, more than half of the studies were conducted in two regions (the United States and the United Kingdom), and there was only one study from Canada. In addition, many more studies focus on intervention practices compared to assessment practices despite assessment being an essential component to develop an appropriate intervention plan. Of note, very few studies provided specific details about the nutrition care encounters (e.g., types of dietary assessment tools used, types of information provided to patients, strategies used to provide the information, length of time spent on the encounter, follow-up on encounters). A consideration for future research in this area is that the standardized Nutrition Care Process (NCP) could be used when thinking about how to approach conducting research on this topic. This framework was developed by the Academy of Nutrition and Dietetics (formerly the American Dietetic Association) [103] and distinguishes nutrition care into four components: Nutrition Assessment, Nutrition Diagnosis, Nutrition Intervention, and Nutrition Monitoring and Evaluation. Use of this framework may be useful for structuring future research in this area.

Several studies did not specifically outline the oral health issue and patient population that was being studied; when studies examined nutrition care practices in oral health professionals, it was assumed that they were referring to oral health (and specifically the dentition and periodontium). Although this assumption is likely relatively safe, a limitation of this review is that through the process of conducting this project, we found evidence that oral health professionals may also provide some nutrition care in other areas such as diabetes [104,105] and obesity/weight management [106,107,108,109]. We felt that the inclusion of these articles that did not mention the specific oral health condition and patient population was important as they represent an important group of studies in this area. 

Another interesting finding from this review was that information on nutrition care practices for oral health (and specifically the dentition and periodontium) was captured using various study designs both in terms of the research methodology (e.g., cross-sectional survey, observation, chart review, qualitative interview) and the overall focus of the articles. In addition, many studies collecting information on nutrition care practices of oral health professionals capture this information as part of a study with a non-nutrition focus such as examining overall practice patterns of oral health professionals (e.g., [66,67]), dental prevention activities (including for dental caries) (e.g., [41,42,55,58,59,77,79]), or infant oral health care (e.g., [36,63,68,70,71,85,87]). However, a few studies focused primarily on diet and oral health (e.g., [35,40,43,75,80,82]). Because diet was often not the focus of the included studies (and instead a subcomponent), one major challenge encountered when conducting this review was that many of the included articles were difficult to locate solely from database searching. As can be seen in Figure 1, many articles were located using other approaches (e.g., citations, hand searching). With diet being such an important component of optimal oral health, researchers conducting work in this area should consider including words related to diet/nutrition in the title, abstract, and keywords of articles to ensure that these studies will be found using database searches. In addition, more studies with a focus on diet and oral health should be conducted to focus on these specific activities, particularly because these activities are complex and multifaceted. 

Overall, most of the articles included in this review found that nutrition care is provided by most oral health professionals at least some of the time. Survey studies and studies capturing self-report data found that provision of nutrition care was more common compared to when it was examined in chart review and observation studies. Some studies included as part of this review provide information on both barriers to providing nutrition care by oral health professionals and factors that affect the provision of these types of services, including the professional type, patient type, and various professional and practice factors. Together, this information suggests that many different factors can influence whether professionals provide this type of service and that there are many barriers that they experience when attempting to provide this type of care. This information is helpful for both dietitians and oral health professionals aspiring to provide this care, as well as managers interested in facilitating this process in different types of practice settings. Capturing this type of information in future studies is important to help optimize the provision of these types of services. 

Dietitians are a professional group possessing the capability to offer substantial support in this area and have the knowledge, expertise, and time to focus on providing nutrition care to individuals who are in need of improving their diet to optimize oral health. Nutrition care by dietitians may also be a solution to address some of the barriers to performing these types of interventions identified by oral health professionals, including time (as they have more time to devote to nutrition care); lack of patient motivation, compliance, and/or interest (as they are trained to provide nutrition care to address these barriers such as motivational interviewing); and lack of knowledge/confidence among oral health professionals in providing this type of care (as dietitians are specifically trained to provide nutrition care). Despite the skillset of dietitians, this review identified only a handful of studies that captured information on nutrition care for oral health provided by dietitians and collaboration between dietitians and oral health professionals. Importantly, within the studies identifying collaboration between dietitians and dental professionals, findings suggest that this activity is uncommon despite various organizations and peer-review articles highlighting the importance of collaboration between these professionals [8,110,111]. In addition, dietitians have identified barriers in this area, including cost, lack of client interest in this area, dietitian confidence in providing support in this area, and concerns about reluctance of oral health professions to provide referrals to dietitians. Finding new ways to stimulate collaboration between the different professions will likely be very important in this area and overcoming barriers in this area is needed. Increased training on this topic for both professionals and students is important to consider. 

There are a few limitations of this review. Although we did our best to locate articles using different strategies and are confident that we were able to locate the vast majority of the research studies in this area that met inclusion criteria, there are likely articles that were missed. Only studies written in English were included; there could have been relevant studies available in other languages that may have been excluded. We also did not assess nutrition care practices regarding oral health by professionals other than oral health professionals and dietitians, and there may be others who also provide these services. In addition, this review did not assess study quality.

## 5. Conclusions

Many different types of studies have captured information on nutrition care practices related to oral health (and specifically dentition and periodontium) in oral health professionals, and very few are available focusing on dietitians. In addition, there are limited data available on the specific details of the care that is provided. Few studies have captured information on interprofessional collaboration between dietitians and oral health professionals. This review article provides insight into how to move this area of research forward.

## Figures and Tables

**Table 1:** MEDLINE search strategy.

**Concept Category #1:** Oral and Dental Health, Dentistry, Oral Health Professionals	**Concept Category #2:** Dietitians, Nutrition Services, Dietetics, Food, Nutrition, Diet	**Concept Category #3:** Professional Practices and Behaviours
Oral Health/OR exp periodontal diseases/OR exp tooth diseases/OR exp Dentition/OR exp Odontogenesis/OR exp dental auxiliaries/OR exp dental staff/OR exp dentists/OR exp faculty, dental/OR exp dentistry/OR exp public health dentistry/OR exp Dental Facilities/OR exp Dental Health Services/OR Dental Records/OR exp societies, dental/OR Licensure, Dental/OR Schools, Dental/OR exp Education, Dental/OR Insurance, Dental/OR exp Economics, Dental/OR dent*.ti,ab,kw. OR tooth.ti,ab,kw. OR teeth.ti,ab,kw.	exp “diet, food, and nutrition”/OR exp Carbohydrates/OR exp sugar alcohols/OR drinking behavior/OR exp feeding behavior/OR exp Feeding Methods/OR Cariogenic Agents/OR Nutritionists/OR exp Nutritional Sciences/OR exp Dietary Services/OR exp Nutrition Therapy/OR nutrition assessment/OR exp Body Weight/OR exp Nutrition Disorders/OR diet*.ti,ab,kw. OR sugar*.ti,ab,kw. OR nutritio*.ti,ab,kw.	“attitude of health personnel”/OR attitude to health/OR health knowledge, attitudes, practice/OR Practice Patterns, Dentists’/OR professional-patient relations/OR professional-family relations/OR interprofessional relations/OR interdisciplinary communication/OR intersectoral collaboration/OR Patient Care Team/OR Professional Practice Gaps/OR exp Professional Practice/OR exp Professional Role/OR practice*.ti,ab,kw. OR interprofessional*.ti,ab,kw. OR interdisciplin*.ti,ab,kw. OR multidisciplin*.ti,ab,kw. OR collaborat*.ti,ab,kw. OR multi-disciplin*.ti,ab,kw. OR inter-disciplin*.ti,ab,kw. OR trans-disciplin*.ti,ab,kw. OR transdisciplin*.ti,ab,kw. OR cross-disciplin*.ti,ab,kw. OR referral*.ti,ab,kw.

**Table 2:** Inclusion and exclusion criteria.

Inclusion Criteria	Exclusion Criteria
Article published in year 2000 or later Peer-reviewed research article Article written in English Study conducted in humans Study conducted in a World Bank high-income country Article reported information on the real-world nutrition care practices (e.g., diet advice, nutrition counselling) (including barriers) of dietitians/nutritionists/WIC personnel and/or oral health professionals to optimize oral health (and specifically the dentition and periodontium). ○ Studies describing the nutrition care practices of oral health professionals without reference to a specific oral health condition were included as they were assumed to be referring to oral health, specifically the dentition/periodontium. Articles that reported practices related to oral health (e.g., tooth brushing counselling) in dietitians/nutritionists/WIC personnel without reference to a specific oral health condition were included as they were assumed to be referring to oral health and specifically dentition/periodontium.	Letters to the editor, commentaries, conference abstracts, thesis documents, grey literature, review articles Information about practices and/or barriers of providing nutrition care reported by students (e.g., nutrition students, dental students, dental hygiene students) Information about nutrition care practices for dietitians/nutritionists/WIC personnel and/or oral health professionals is combined with other dental prevention activities (and cannot be isolated) Information about nutrition care practices of dietitians/nutritionists/WIC personnel and/or oral health professionals is combined with another profession (e.g., nurses, physicians) Information on nutrition care practices provided by dietitians/nutritionists/WIC personnel and/or oral health professionals from the patient perspective Information about practices regarding fluoride supplements, sugar alcohols, herbal supplements, homeopathy, and alcohol by oral health professionals Information about diabetes, obesity, cardiovascular disease, hypertension screening/management by oral health professionals Information about body weight and blood glucose screening/monitoring by oral health professionals Information about how dietitians/nutritionists/WIC personnel and/or oral health professionals would treat hypothetical patient case scenarios/studies

WIC: Special Supplemental Nutrition Program for Women, Infants, and Children.

**Table 3:** Assessment practice information reported in the included articles.

Study Authors, Year, and Country	Sample Size *	Professional Type	Population and/or Concern of Focus	General/Unspecific Assessment Practices (e.g., Dietary Analysis)	Use of Specific Assessment Tools, Strategies, or Practices (e.g., Food Records)	Inquiries about Specific Patient Behaviours or Concerns (e.g., Bottle Use, Cariogenic Food Consumption)	Barriers (or Reasons for Lack of Use) Regarding Assessment Practices
SURVEY STUDIES							
Oral Health Professionals							
Roshan et al., 2003 [33]; UK	687	general dental practitioners; community dental officers	children	(X)^Practice/Prof^			
Hawkins and Locker, 2005 [34]; Canada	672	general dentists	older adults		X	X^Practice/Prof^	
Sajnani-Oommen et al., 2006 [35]; USA	180	pediatric dentists	children		X		
Bubna et al., 2012 [36]; USA	554	pediatric dentists	infants	(X)			
Clovis et al., 2012 [37]; USA	540	dental hygienists	children; dental caries			X^Practice/Prof^	
Mulic et al., 2012 [38]; Norway	705	public dental health service dentists	adults; dental erosive wear	X	X		
Garton and Ford, 2013 [39]; Australia	638	dentists (including specialists)	root caries	X			X^Practice/Prof^
Sim et al., 2014 [40]; USA	86	pediatric dentists	infants and toddlers; dental caries			X	
Yusuf et al., 2016 [41]; UK	164	National Health Service general dental practitioners		X^Practice/Prof^			
Widström et al., 2016 [42]; Norway	215 dentists; 166 dental hygienists	dentists; dental hygienists in public dental service	public dental service clients—children and adults	X^Professional Type; Patient^			
Arheiam et al., 2016 [43]; UK	250	general dental practitioners	children; adults		X^Practice/Prof; Patient^		X^Patient^
Halawany et al., 2017 [44]; Saudi Arabia	108	pediatric dentists	children; dental caries	X			
Dima et al., 2018 [45]; Taiwan	196	general dentists; pediatric dentists	children; early childhood caries			X^Professional Type^	
Mulic et al., 2018 [46]; Iceland	153	dentists	dental erosive wear	X	X		
Kangasmaa et al., 2021 [47]; Finland	814	general dentists; dental specialists	erosive tooth wear	X^Practice/Prof; Professional Type^			
Mortensen et al., 2021 [48]; Denmark	419	dentists	erosive tooth wear	X	X		
**Dietitians/Nutritionists**							
Fuller et al., 2014 [49]; USA	159	WIC program personnel	WIC clients; early childhood caries	X^Practice/Prof^			
Gold and Tomar, 2016 [50]; USA	9	WIC nutritionists	WIC clients	X	X	X	
Fernandez et al., 2017 [51]; USA	36	dietitians who completed a pediatric dentistry internship rotation		X^Patient^			
**CHART REVIEW STUDIES**							
**Oral Health Professionals**							
Sarmadi et al., 2009 [52]; Sweden	432	public dental service (dentists and dental hygienists)	children 6–19 years at high risk of developing dental caries	X^Patient^			
O’Toole et al., 2018 [53]; UK	285	dentists	adults; tooth wear	X^Practice/Prof^			
**QUALITATIVE INTERVIEW STUDIES**							
**Oral Health Professionals**							
Threlfall et al., 2007 [54]; UK	93	general dental practitioners	children; dental caries	X^Patient^	X		

* Sample size for survey studies and qualitative interview studies refers to number of professionals included; sample size for chart review studies refers to the number of patients included. WIC: Special Supplemental Nutrition Program for Women, Infants, and Children. (X) = study measure included both assessment and intervention practices combined together (e.g., analyzed diets and gave dietary advice). Patient = examine differences in assessment practices in different types of patients (e.g., children vs. adults, severe dental caries vs. not). Professional Type = examine differences in assessment practices in different types of professionals (e.g., general dentists vs. pediatric dentists, dentists vs. dental hygienists). Practice/Prof = examine differences in assessment practices for different practice characteristics (e.g., public vs. private, rural practice vs. urban practice) and/or health professional characteristics (e.g., demographics, education, attitudes).

**Table 4:** Intervention practice information reported in the included articles.

Study Authors, Year, and Country	Sample Size *	Professional Type	Population and/or Concern of Focus	General/Unspecific Intervention Practices (e.g., Nutrition Counselling, Diet Advice)	Types of Resources/ Strategies Used	Information Provided to Patients	Barriers Regarding Intervention Practices	Referrals/Collaboration between Dentistry and Nutrition/Dietetics
SURVEY STUDIES								
Oral Health Professionals								
Chisick et al., 2000 [55]; USA	606	full-time military or civilian dentists in the Army Dental Care System		X	X	X		X
Anderson et al., 2002 [56]; Wales	568	dentists; dental hygienists; dental therapists				X		
Roshan et al., 2003 [33]; UK	687	general dental practitioners; community dental officers	children	(X)^Practice/Prof^				
Dugmore and Rock, 2003 [57]; UK	227	general and community dental practitioners	children; tooth erosion	X^Patient^		X^Patient^		
Freeman et al., 2005 [58]; Northern Ireland	128 practices	general dental practices	dental caries	X				
Wang, 2005 [59]; Norway	199 (1995); 210 (2004)	dental hygienists from public dental service	children; dental caries	X^Patient^				
Huang et al., 2006 [60]; USA	111	orthodontists	children; dental caries	X^Practice/Prof^	X	X	X	X
Sajnani-Oommen et al., 2006 [35]; USA	180	pediatric dentists	children	X^Practice/Prof^		X		X
Dyer and Robinson, 2006 [61]; UK	166	principal dentists		X			X^Professional Type^	
Trueblood et al., 2008 [62]; USA	127	pediatric dentists	children; dental caries	X				
Brickhouse et al., 2008 [63]; USA	~221	general dentists; pediatric dentists	infants	X^Professional Type^		X^Professional Type^		
Kelly and Moynihan, 2008 [64]; UK	879	dentists; dental hygienists; other occupations or specialties	periodontal disease	X		X	X	
Csikar et al., 2009 [65]; UK	386	dental practitioners		X^Practice/Prof^				
Satur et al., 2009 [66]; Australia	59	dental therapists		X^Practice/Prof^				
Tseveenjav et al., 2009 [67]; Finland and Norway	682	dental hygienists		X^Practice/Prof^				
Malcheff et al., 2009 [68]; USA	2157	pediatric dentists	infants	X				
Manski and Parker, 2010 [69]; USA	308	dental hygienists	children; early childhood caries	X^Practice/Prof^				
Salama and Kebriaei, 2010 [70]; USA	371	general dentists	infants	X				
Ananaba et al., 2010 [71]; USA	2294	general dentists; pediatric dentists	infants	X^Professional Type;^ ^Practice/Prof^				
Cunha-Cruz et al., 2010 [72]; USA	209	dentists	dentin hypersensitivity	X				
Bubna et al., 2012 [36]; USA	554	pediatric dentists	infants	(X)				
Lee et al., 2012 [73]; USA	1779	pediatric dentists	children; dental caries	X^Practice/Prof^				
Kakudate et al., 2012 [74]; Yokoyama et al., 2013 [75]; Yokoyama et al., 2013 [76]; Japan	189	dentists		X^Practice/Prof^				
Hussein et al., 2013 [77]; Germany	640	dentists		X^Practice/Prof^				
Sim et al., 2014 [40]; USA	86	pediatric dentists	infants and toddlers; dental caries	X^Practice/Prof^			X	
Gnich et al., 2014 [78]; Scotland	174	dental nurses	children	X^Patient; Practice/Prof^				
Yusuf et al., 2015 [79]; Yusuf et al., 2016 [41]; UK	164	National Health Service general dental practitioners		X^Practice/Prof^				
Arheiam et al., 2016 [43]; UK	250	general dental practitioners		X	X			
Hayes et al., 2016 [80]; Australia	426	dental hygienists; oral health therapists					X	
Baatsch et al., 2017 [81]; Germany	250	dentists						X^Practice/Prof^
Hayes et al., 2017 [82]; Australia	41	dentists; dental hygienists; oral health therapists		X^Professional Type^			X^Professional Type^	
Wright and Casamass-imo, 2017 [83]; USA	1615	pediatric dentists; pediatric dental residents	children; sugar sweetened beverages	X^Practice/Prof^	X	X	X	X
Cole et al., 2018 [84]; USA	919	dental hygienists	children	X^Practice/Prof^	X^Practice/Prof^	X		X^Practice/Prof^
Djokic et al., 2019 [85]; Ireland	467	pediatric dentists; nonpediatric dentists	infants	X^Professional Type^				
Aziz et al., 2020 [86]; New Zealand	325	general dentists		X^Practice/Prof^				
Bakhurji et al., 2021 [87]; Saudi Arabia	335	general dentists; pediatric dentists	infants	X^Professional Type^		X^Professional Type^		
**Dietitians/Nutritionists**								
Shick et al., 2005 [88]; USA	324	WIC nutritionists	WIC clients					X^Patient; Practice/Prof^
Butani et al., 2006 [89]; USA	126	WIC providers	WIC clients	X^Practice/Prof^				
Fuller et al., 2014 [49]; USA	159	WIC personnel	WIC clients			X^Practice/Prof^		X^Practice/Prof^
Gold and Tomar, 2016 [50]; USA	9	WIC nutritionists	WIC clients			X		X
Fernandez et al., 2017 [51]; USA	36	dietitians who completed a pediatric dentistry internship rotation		X			X	X
**CHART REVIEW STUDIES**								
**Oral Health Professionals**								
Kärkkäinen et al., 2001 [90]; Finland	267 in 1992; 590 in 1995	public dental service	children 12 years and 15 years	X^Patient^^; Practice/Prof^				
Tickle et al., 2003 [91]; UK	677	general dental practices (*n* = 50)	children who regularly attended dental care and have a history of interproximal caries in primary molars	X^Patient^				
Nihtilä and Widström, 2009 [92]; Finland	466	public dental service	children and adolescents	X^Patient^				
Wang and Aspelund, 2010 [93]; Norway	576	public dental service (20 clinicians in 16 public dental service clinics)	children and adolescents 3–18 years	X^Patient^				
Sarmadi et al., 2011 [94]; Sweden	432	public dental service (dentists and dental hygienists)	children 6–19 years at high risk of developing dental caries	X^Patient^				
Masoe et al., 2014 [95]; Australia	29,599	public dental service (oral health therapists)	adolescents 12–18 years	X^Patient^^; Practice/Prof^				
Raindi et al., 2015 [96]; UK		general dental practice	periodontal disease	X				
Skinner et al., 2016 [97]; Australia	~26,000 to ~31,000 per year	public dental service	adolescents 12–17 years	X^Patient^				
**OBSERVATION, CHART REVIEW, AND SURVEY STUDY**								
**Oral Health Professionals**								
Demko et al., 2008 [98]; Wotman et al., 2010 [99]; USA	3751 patient visits in 120 general dental practices	dentists, dental hygienists		X^Professional Type^				
**QUALITATIVE INTERVIEW AND/OR FOCUS GROUP STUDIES**								
**Oral Health Professionals and Dietitians/Nutritionists**								
Threlfall et al., 2007 [100]; Threlfall et al., 2007 [54]; UK	93	general dental practitioners	children; dental caries	X^Patient; Practice/Prof^	X	X^Patient^	X	
Cashmore et al., 2011 [101]; Australia	10	dental assistants, dental therapists, pediatric dental specialist, regional co-ordinator of oral health promotion	children waiting for surgery for treatment of severe dental caries				X	
Ong et al., 2015 [102]; Hong Kong	23	dentists, dietitians						X

* Sample size for survey studies and qualitative interview and/or focus group studies refers to number of professionals included; sample size for chart review and observational studies refers to the number of patients included. WIC: Special Supplemental Nutrition Program for Women, Infants, and Children. (X) = study measure included both assessment and intervention practices combined together (e.g., analyzed diets and gave dietary advice). Patient = examine differences in intervention practices in different types of patients (e.g., children vs. adults, severe dental caries vs. not). Professional Type = examine differences in intervention practices in different types of professionals (e.g., general dentists vs. pediatric dentists, dentists vs. dental hygienists). Practice/Prof = examine differences in intervention practices for different practice characteristics (e.g., public vs. private, rural practice vs. urban practice) and/or health professional characteristics (e.g., demographics, education, attitudes).
